# Structural systems pharmacology: A framework for integrating metabolic network and structure-based virtual screening for drug discovery against bacteria

**DOI:** 10.1371/journal.pone.0261267

**Published:** 2021-12-14

**Authors:** Elmira Nazarshodeh, Sayed-Amir Marashi, Sajjad Gharaghani

**Affiliations:** 1 Laboratory of Bioinformatics and Drug Design (LBD), Institute of Biochemistry and Biophysics, University of Tehran, Tehran, Iran; 2 Department of Biotechnology, College of Science, University of Tehran, Tehran, Iran; The University of Alabama in Huntsville, UNITED STATES

## Abstract

Advances in genome-scale metabolic models (GEMs) and computational drug discovery have caused the identification of drug targets at the system-level and inhibitors to combat bacterial infection and drug resistance. Here we report a structural systems pharmacology framework that integrates the GEM and structure-based virtual screening (SBVS) method to identify drugs effective for *Escherichia coli* infection. The most complete genome-scale metabolic reconstruction integrated with protein structures (GEM-PRO) of *E*. *coli*, iML1515_GP, and FDA-approved drugs have been used. FBA was performed to predict drug targets *in silico*. The 195 essential genes were predicted in the rich medium. The subsystems in which a significant number of these genes are involved are cofactor, lipopolysaccharide (LPS) biosynthesis that are necessary for cell growth. Therefore, some proteins encoded by these genes are responsible for the biosynthesis and transport of LPS which is the first line of defense against threats. So, these proteins can be potential drug targets. The enzymes with experimental structure and cognate ligands were selected as final drug targets for performing the SBVS method. Finally, we have suggested those drugs that have good interaction with the selected proteins as drug repositioning cases. Also, the suggested molecules could be promising lead compounds. This framework may be helpful to fill the gap between genomics and drug discovery. Results show this framework suggests novel antibacterials that can be subjected to experimental testing soon and it can be suitable for other pathogens.

## 1. Introduction

The experimental drug discovery process is expensive, resource-intensive, and time-consuming. Computational drug discovery approaches facilitate the identification and evaluation of potential drug molecules. Therefore, these methods can be an effective plan to accelerate drug development and reduce costs. Such methods are essential in the early stage of drug discovery [1,2]. Furthermore, the drug resistance of pathogens in humans is a critical emerging issue nowadays. Therefore, finding new drug targets, and consequently, new anti-infective agents are necessary. On the other hand, due to the complexity of infectious diseases, effective therapeutic strategies are required. Therefore, identifying multiple druggable targets, *e*.*g*., by systems biology approaches, is preferred to those approaches which find single-targets.

In some of the previous studies, conserved genes in pathogens obtained by comparative genomic analyses were assumed as drug targets [3]. However, genome-scale metabolic models (GEMs) can provide more biological information, and analyzing the metabolic networks as a system-oriented approach will accelerate the process of finding essential drug targets [4,5]. SBVS is a computational approach that searches a set of ligands to discover potential active molecules for a protein. There is no need for physically existing molecules and this is an advantage of virtual screening methods [6].

In this work, we present a structural systems pharmacology framework to identify drug-target [4] interactions by coupling analyzing a genome-scale metabolic model integrated with protein structures (GEM-PRO) and a SBVS method. This framework uses the advantages of both approaches and is valuable for the drug discovery field. This strategy is able to identify drug targets on the system-level aspect and then drugs for their inhibition simultaneously. Here, we have focused on the gram-negative bacterium *Escherichia coli* K-12 MG1655 as a case. Intestinal pathogenic *E*. *coli* (IPEC) causes intestinal infection, including diarrhea or dysentery. Enteropathogenic *E*. *coli* (EPEC) is a subgroup of IPEC and *E*. *coli* K-12 is a well-known model for EPEC strains [7]. *E*. *coli* K-12 is the most completely characterized organism and a laboratory strain.

We used GEM-PRO of *E*. *coli* for extraction of essential genes for the growth as druggable targets, and then, we identified potential modulators of the targets via a SBVS method. We applied our computational strategy for doing drug repurposing against *E*. *coli* which can accelerate drug discovery efforts. We anticipate this framework can be applied for other bacterial pathogens with validated GEM to inhibit their caused infection.

## 2. Material and methods

### 2.1. Genome-scale metabolic network model

Genome-scale metabolic models (GEMs) are fundamental and widely-trusted tools in systems biology to study metabolism *in silico*. The GEMs are shown to be useful for data interpretation and physiological predictions [8,9]. We used the genome-scale metabolic reconstruction iML1515 and iML1428-iso (a context-specific version) of *E*. *coli* strain K-12 substrain MG1655 (Taxonomy ID: 83333) integrated with proteins (GEM-PRO) [10]. These reconstructions have the most comprehensive information to date for *E*. *coli* metabolism. The former model, iML1515, includes related protein structures and integrates systems and structural biology. The context-specific model iML1428-iso considers only dominant isozymes of iML1515 reactions, that is, the isozymes with higher expression in glucose M9 medium. This model is more accurate in predicting gene knockout. Therefore, iML1428-iso was used in subsequent analysis. The model iML1428-iso contains 1429 genes, 2712 reactions, 1877 metabolites. We checked the model validation with MEMOTE (Metabolic Model Tests), a standardized testing suite for GEMs [11]. To obtain the growth rate and perform the subsequent metabolic simulations, a rich medium was considered, to simulate the human body conditions. The components of the defined medium are listed in Supplementary S1 Table.

### 2.2. Identification of potential drug targets

For constraint-based modeling of metabolic fluxes in GEMs, we used COBRApy v 0.16.0 [12], with ‘glpk’ as the linear programming solver [13]. To predict the essentiality of metabolic genes, single gene deletion simulations were done using flux balance analysis (FBA) by considering the gene-protein-reaction (GPR) relationships. For each gene, the flux of its corresponding reaction was constrained to zero, and then, FBA was used to study its effect on biomass production rate [14]. The wild-type biomass reaction in the model (*i*.*e*., BIOMASS_Ec_iML1428_WT_75p37M) was set as the objective function of FBA. The rich medium was considered for the simulations by adding some of the exchange reactions to the model using the lower bound set equal to -0.1 mmol/gDW/h. A gene is considered essential for the cell if its knock out decreases the growth rate to less than five percent of its maximum value.

### 2.3. Subsystems and GO terms of essential genes

The subsystems associated with the essential genes were identified [10]. Additionally, the essential genes were enriched with gene ontology (GO) terms from the UniProt Knowledgebase (UniProtKB) [15]. GO terms describe the biological role of genes from three different aspects, namely Biological Process (BP), Molecular Function (MF), and Cellular Component (CC) [16]. CC terms determine which genes are associated with the cell membrane. The relevance of the essential genes and their encoded products (*i*.*e*., the potential drug targets) to fight against the bacterium is investigated by analyzing their subsystems and associated GO terms.

### 2.4. Exclusion of identified essential genes with human homologs

To choose potential drug targets from the list of essential genes of *E*. *coli*, those genes that have at least a human homolog are excluded from the list. To achieve this goal, we used the PathoSystems Resource Integration Center (PATRIC) (https://www.patricbrc.org) [17]. We used the information about human homologs for the *E*. *coli* K12 MG1655 obtained by BLASTP in the PATRIC database.

### 2.5. Identifying 3D structures and their co-crystallized ligands for the essential gene product

For linking the metabolic network to the 3D structures of its proteins, we utilized *ssbio*. The *ssbio* package provides a framework to work with structural information of proteins in genome-scale network reconstructions [18]. Representative structures were mapped to each identified essential gene. They were selected based on QC/QA criteria such as resolution, number of mutations, and completeness [10]. UniProt IDs are obtained from the UniProt metadata and mapped to each gene. The chemical compound (cognate ligands) information was obtained from the Ligand Expo database [19] and Protein Data Bank (PDB) metadata of the GEM-PRO. We mapped information of the bound ligands to the essential genes using this extracted data. Ligand Expo database provides chemical and structural information about small molecules within the structures of the PDB. The chains of protein structures with bound ligands needed to run the next step SBVS method are detected using the PDB metadata.

After excluding the essential genes that have human homologs, some further filters were applied to select only the most informative protein-ligand complexes. Briefly, the genes with no experimental structure were excluded. Among the essential genes, 117 (62%) of them have experimentally resolved structures. Using information from Ligand Expo database that has 123871 pairs of protein-ligands, the experimental structures with no co-crystallized ligands were also removed, as the bound ligand is needed to describe a protein binding pocket for performing the SBVS in the next step. So, we considered 103 genes whose protein structures have bound ligands (54.35%). Additionally, the essential genes that have structures with only a metal ion as the bound ligand were excluded. Moreover, the semi-manually curated BioLiP database was used to remove biologically irrelevant ligand-protein interactions, which are related to those molecules that are added merely for obtaining protein crystals. After removing proteins with irrelevant bound ligands, 70 essential genes remained. The protein products of some of these 70 genes have more than one bound ligand, and hence, 92 protein-ligand pairs were considered for performing PLPS2.

Those structures that could pass the above filters were used in the SBVS step. We downloaded the structure files of these shortlisted proteins from the RCSB Protein Data Bank (PDB) website.

### 2.6. FDA-approved drugs

The data set used for doing SBVS is the 3D structure of FDA-approved drugs downloaded from the ZINC15 library [20]. ZINC15 is a free database of commercially-available, ready-to-dock, and 3D compounds for virtual screening. Open Babel, an open-source chemistry toolkit, was applied to find and remove potentially redundant molecules from the data set [21]. Finally, the data set of 1404 MOL2 files was used in the SBVS step.

### 2.7. Structure-based virtual screening to rank FDA-approved drugs against drug targets

We generated multiple conformations (maximum of 50 conformers) for each molecule using the Confab [22] option of Open Babel to consider the molecule flexibility. Confab needs a 3D structure of a molecule as the input file and generates diverse low-energy conformers. A default RMSD cutoff of 0.5 Å was set in this step.

Structure-based virtual screening was performed with FDA-approved drugs for identified drug targets of *E*. *coli* using PL-PatchSurfer2 (PLPS2) [23]. Further inspection was done to possibly select the most potential and pathogen-specific compounds that could inhibit more than one drug target at once. These compounds are proposed to be used in polypharmacology cases. To apply PLPS2, a protein structure file (PDB) with a co-crystallized ligand bound to it for identification of a binding pocket, and a set of small molecule files (MOL2) are needed. PLPS2 finds complementarities on surfaces between binding pockets and conformers of molecules. First, after detecting the binding pocket, the separation of the bound ligand from its target is automatically done for all targets. The molecular surface of the targets and the conformations of molecules are created by the Adaptive Poisson-Boltzmann Solver (APBS) software package [24]. The input file for APBS is prepared by PDB2PQR software, via converting the PDB file to PQR format by assigning atom charge and radius information [25]. After that, the generated surfaces are ‘sliced’ into overlapping local patches to assess the local matching of the target pocket and the molecule conformation. For the surfaces of the patches, four features, namely shape, electrostatic potential (calculated using APBS), atom-based hydrophobicity (calculated using XLOGP3 method) [26], and hydrogen-bond acceptor/donor are represented with three-dimensional Zernike descriptors (3DZDs) [27]. 3DZD is a vector representation of a mathematical 3D function in Euclidean space, and it is invariant to rotation. SSIC files are generated with the information of patches for targets and ligands. The number of patches, the coordinates of the center of patches, and 3DZDs of four features are in the SSIC files. Then, to extract compatible patch pairs, a comparison between patches of a binding pocket and a molecule conformation is performed using the Auction algorithm. Then, identified complementaries are estimated using a score. The score ranks ligands against each drug target. To calculate the score for each molecule, the Boltzmann-Weighted Score (BS) has been used [28,29]. To sort molecules, BS uses a weighted average of scores of all molecule conformers. The performance of PLPS2 has been examined with four data sets. This SBVS approach works faster than the other available common methods, including AutoDock Vina, DOCK6, and ROCS. It has been shown that the surface patch representation [30] enhances tolerance to conformation changes of targets, and this is an advantage of PLPS2.

This approach ranks FDA-approved drugs against each identified essential target. Therefore, the best-ranked molecules were obtained for each target. Besides, the best-ranked molecules that have good interaction with more than one target are proposed as potential polypharmacology cases. The polypharmacology opportunities were determined based on three different strategies. In the first method, the drugs in the only top (first) rank of all targets were checked. Also, the top five ranks and 1-percentile were considered in the second and third methods, respectively. In the two latter methods, we checked molecules in other top ranks to prevent the loss of the possibly effective molecules. In the 1-percentile approach, we divide the distance between the best and the worst BS values into 100 equal parts for each target, and then, we take the ligands (drugs) that are in the one percent of this distance (their scores are better than the 1-percentile). Then, 30 ligands that have been filtered for more proteins were selected.

Also, agglomerative hierarchical clustering dendrograms are shown on the heat maps for both targets and drug molecules via seaborn [31] which is a Python data visualization library based on matplotlib [32]. The individual data points are as one cluster and in each iteration combines using a bottom-up approach. The method used for calculating the distance between the newly formed clusters is “average” and the metric to compute the distance between m points is “Euclidean distance” (2-norm). Score values of the final selected ligands are normalized between 0 and 1 for each essential target. Then, rescaling of the scores is done with a linear function according to the following formula based on each row (each target):

$$Z_{i}=\frac{max\left(x\right)-x_{i}}{max\left(x\right)-\text{min}\;\left(x\right)}$$ (1)

Where *x*_*i*_ is the BS value of the molecule, max (*x*) is the maximum of BS among molecules for each target, and min (*x*) is the minimum of BS among molecules for each target. Therefore, the best ligand for each target obtains the highest normalized score. Performing PLPS2 and creating all input files needed for different steps were carried out automatically using Python programming.

### 2.8. ATC-code of the selected drugs

We inspected the characteristics of the final shortlisted molecules which were predicted to stop the growth of the bacterium in the DrugBank [33]. DrugBank is a free database with drug information, their mechanisms, interactions, and targets. Anatomical Therapeutic Chemical (ATC) code of selected top ligands was checked from the World Health Organization (WHO) Guidelines 2020. The drug’s ATC Classification System classifies the active ingredients of drugs in a hierarchy with five different levels. We investigated whether our shortlisted drugs are anti-infectives.

## 3. Results and discussion

### 3.1. Identification of essential metabolic genes as potential drug targets

We used iML1428, the context-specific genome-scale metabolic network of *E*. *coli* K-12 integrated with proteins (GEM-PRO), to determine the maximum growth rates in minimal and rich media. Then, we identified essential genes for the growth of the bacterium. We simulated growth on a rich medium to simulate the human body condition [14,34]. The rich medium assumption was applied by opening the flux of exchange reactions of those metabolites that exist in the yeast extract [35]. The availability of nutrients has a major impact on metabolic fluxes.

We used flux variability analysis (FVA) for identifying blocked reactions. From the list of all 2712 metabolic reactions in iML1428-iso, there are 260 universally blocked reactions (9.58%), which cannot carry any nonzero flux while all model boundaries are unconstrained. Also, we found 968 (35.69%) and 895 (33.00%) blocked reactions in minimal and rich media, respectively.

The wild-type biomass reaction (BIOMASS_Ec_iML1428_WT_75p37M) was set as the objective function. The ultimate goal of this study is to find drugs that can prevent bacterial growth, and therefore, biomass objective function [36] is appropriate for predicting the potential drug targets [37,38]. The growth was zero in the minimal medium (glc lb = -10 mmol/gDW/h) for the organism. To find the reason, we validated the model by MEMOTE. According to MEMOTE report, when the model is simulated on the provided minimal medium, one precursor (adenosylcobalamin [’adocbl_c’]) of biomass reaction cannot be produced. This metabolite is one of the biologically active forms of vitamin B12. To solve this problem, we set the flux lower bound of adenosylcobalamin (EX_adocbl_e) exchange reaction to -0.1 mmol/gDW/h. Finally, the optimization succeeded and the aerobic growth rates were 0.880 1/h and 1.065 1/h by FBA in the minimal and rich media, respectively.

In the next step, to identify the essential genes for growth, single-gene knockout simulations were done using the FBA method in a rich medium. Firstly, each gene of the model is knocked out, and the maximum flux value through the objective reaction is calculated by FBA. The flux values smaller than 10^−8^ are considered zero, as they are presumably originated from computational numerical errors. Then, we selected those genes whose knocking out results in decreased growth or no growth phenotype. More precisely, those genes whose knockout make the growth rate to decrease to <5% of the maximum growth rate (i.e., <0.053 1/h) were chosen. Finally, we identified 195 essential metabolic genes for growth in the simulated rich medium using FBA method, which comprises 10.7% of genes in the network. The products of these genes were considered potential targets for drug discovery. The list of essential genes and their UniProt IDs (i.e., the potential drug targets) is presented in Supplementary S1 File.

### 3.2. Subsystems and GO terms of the essential genes

We investigated the subsystems/pathways of identified essential genes. The majority of these genes were found to be involved in the ‘Cofactor and Prosthetic Group Biosynthesis’ (73 genes), ‘Lipopolysaccharide Biosynthesis/Recycling’ (38 genes), ‘Cell Envelope Biosynthesis’ (18 genes), and ‘Purine and Pyrimidine Biosynthesis’ (16 genes) pathways. These subsystems are obviously important for bacterial growth.

As the most identified essential genes are in the “Cofactor and Prosthetic Group Biosynthesis” subsystem, we have provided their description and BP GO terms in Supplementary S2 Table. Cofactors have an important role in metabolism. Therefore, genes involved in the biosynthesis process of cofactors could be potential drug targets. According to Supplementary S2 Table, "NAD salvage" and "de novo NAD biosynthetic" are the biological processes of some identified essential genes. NAD cofactor is needed in some biological processes of prokaryotes like redox balance and energy metabolism [39]. On the other hand, some enzymes use NAD as a substrate in processes like DNA repair and degrade it [40]. Therefore, NAD biosynthetic process is required and could provide drug targets to fight against bacteria [39]. As well, genes involved in the coenzyme A and FAD biosynthetic process could be antibacterial targets [41]. Also, vitamins that are organic cofactors, their biosynthetic process are potential drug targets. For example, the biosynthetic pathway of folic acid is a useful target for sulfonamide antibiotics. Besides, the active form of thiamin (thiamin diphosphate) is a vital cofactor for organisms and is necessary for the activity of branched-chain amino acid metabolic enzymes [42,43]. As we can see in Supplementary S2 Table, some of the predicted essential genes are in the biosynthetic process of cobalamin (vitamin B12), thiamine (vitamin B1), riboflavin (vitamin B2), folic acid (vitamin B9), and menaquinone (vitamin K2).

Cell-membrane-related enzymes could be good options for better drug accessibility to stop or slow down the growth of pathogens [44]. Membrane-related essential genes and their membrane-related CC GO terms are shown in Table 1.

**Table 1:** Membrane-related essential genes.

Essential genes	UniProt IDs	Gene description	Membrane-related CC GO term IDs	GO Terms
b4262	P0ADC6	lipopolysaccharide transport system protein LptG	GO:0005886	Plasma membrane
			GO:0043190	ATP-binding cassette (ABC) transporter complex
			GO:0016021	Integral component of membrane
			GO:1990351	Transporter complex
b3201	P0A9V1	lipopolysaccharide transport system ATP binding protein LptB	GO:0043190	ATP-binding cassette (ABC) transporter complex
			GO:0005886	Plasma membrane
			GO:1990351	Transporter complex
b3843	P0AAB4	3-octaprenyl-4-hydroxybenzoate decarboxylase	GO:0005886	Plasma membrane
b4177	P0A7D4	adenylosuccinate synthetase	GO:0016020	Membrane
b0182	P10441	lipid A disaccharide synthase	GO:0019897	Extrinsic component of plasma membrane
b3619	P67910	ADP-L-glycero-D-mannoheptose 6-epimerase	GO:0016020	Membrane
b0586	P11454	apo-serine activating enzyme	GO:0005886	Plasma membrane
b0594	P10378	2,3-dihydroxybenzoate-AMP ligase	GO:0016020	Membrane
b0635	P0AD65	peptidoglycan DD-transpeptidase MrdA	GO:0005887	Integral component of plasma membrane
b0158	P37028	vitamin B12 ABC transporter periplasmic binding protein	GO:0030288	Outer membrane-bounded periplasmic space
b2476	P0A7D7	phosphoribosylaminoimidazole-succinocarboxamide synthase	GO:0016020	Membrane
b3966	P06129	cobalamin outer membrane transporter	GO:0016021	Integral component of membrane
			GO:0031230	Intrinsic component of cell outer membrane
			GO:0046930	Pore complex

**Fig 1:**
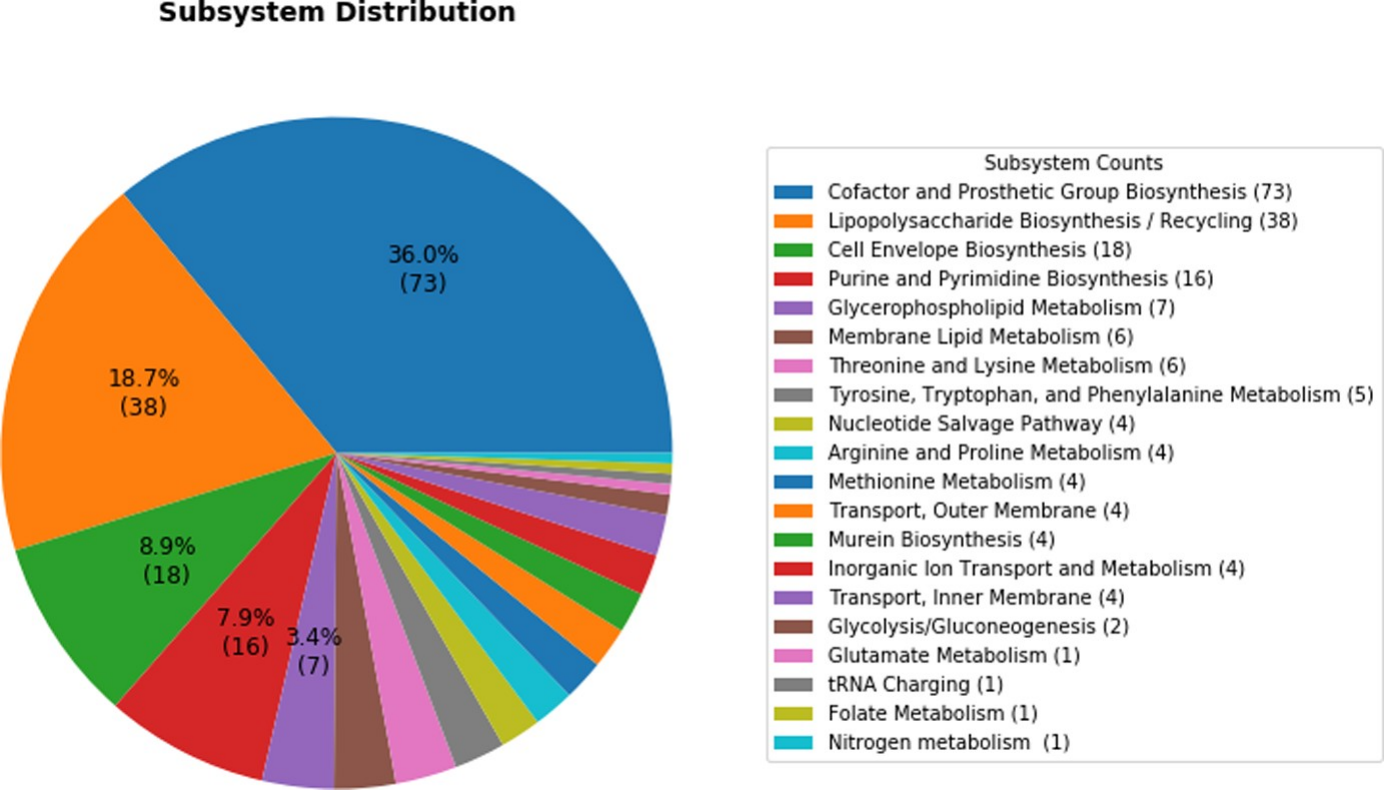
Subsystem distribution of 195 essential genes associated with their frequency.

On the other hand, analysis of the biological processes in which these genes are involved showed that ‘cell wall organization’, ‘lipopolysaccharide biosynthesis’, ‘lipopolysaccharide transport’, and ‘peptidoglycan biosynthetic process’ are the enriched GO terms. The rigid cell wall of Gram-negative bacteria is protection against osmotic lysis. Furthermore, the cell surface of the bacteria composed of LPS, known as endotoxin, provides the first line of protection against antibiotics and other harmful agents [45,46]. Besides, LPS is synthesized in the inner membrane of the cell and is transported to the outer membrane by transporter targets. LPS doesn’t allow antibiotics to enter the cell by creating a barrier and makes bacteria resistant to many antibiotics [47,48]. Therefore, the products of these genes related to cell wall and biosynthesis and transportation of LPS have a high level of importance for bacterial growth and survival [49,50]. Bacterial cell wall compounds are good potential drug target opportunities for killing bacteria or overcoming drug resistance [45,46,51,52]. Fig 1 provides a complete overview of the subsystem distribution of 195 essential genes.

### 3.3. Exclusion of identified essential genes having human homologs

To avoid any probable interference with normal human functions, in the next step, those essential genes that have a human homolog were excluded from the initial list of potential genes [14]. According to the PATRIC platform, there are 54 human homologs for the complete reference genome of *E*. *coli* K-12 MG1655. We obtained UniProt IDs of these genes using their PATRIC IDs and the Retrieve/ID mapping tool of the UniProt website (https://www.uniprot.org/uploadlists/). Then, the set of these 54 human homologs was compared to the set of the 195 essential genes of *E*. *coli*, to find the common genes. Four essential genes with human homologs are listed in Table 2.

**Table 2:** Essential genes with human homologs.

Locus tag	UniProt ID	Gene symbol	Human homolog	EC number
b2530	P0A6B7	iscS	NFS1 (cysteine desulfurase)	2.8.1.7
b2827	P15640	thyA	thyA (Thymidylate synthase)	2.1.1.45
b4005	P15640	purD	GART (Phosphoribosylamine-glycine ligase)	6.3.4.13
b2942	P0A817	metK	MAT2A (methionine adenosyltransferase 2A)	2.5.1.6

### 3.4. Identifying structures and their co-crystallized ligands for each essential gene product

We utilized *E*. *coli* GEM-PRO (iML1515_GP) to obtain representative structures of the 191 essential genes using *ssbio* for performing SBVS. Information about co-crystallized ligands is obtained from the Ligand Expo database and mapped to the essential genes. For all drug targets, the chains with bound ligands are identified from the metadata incorporated in iML1515_GP, using *ssbio*. To perform the SBVS, a ligand-binding pocket is needed and the mentioned filters for the selection of the most informative protein-ligand complexes were applied. At last, 70 essential genes remained. The structures that can succeed in these filtrations are used in the SBVS step. The protein products of some of these 70 essential genes have more than one bound ligand, and hence, 92 protein-ligand pairs are considered for performing PLPS2. Finally, we downloaded the PDB structure of these shortlisted proteins. The complete list of the essential genes and their related information are presented in Supplementary S2 File.

### 3.5. Structure-based virtual screening

We identified essential targets in the previous steps. We ranked the ligands (FDA-approved drugs) against each target by running PLPS2 as an SBVS method. PLPS2 generates a molecular surface for the proteins and molecule conformations using APBS. To investigate the level of matching between a binding pocket and a molecule conformation, generated surfaces are divided into multiple patches. Shape, electrostatic potential, atom-based hydrophobicity, and hydrogen-bonding are represented for the patches with 3DZDs [30]. SSIC files with information about patches are generated for each protein and its ligands. After that, detected compatible pairs of patches are ranked using BS. The ranked ligands against each identified target associated with their score values are shown in Supplementary S3 File. Each row of the matrix is related to ranked ligands for each essential target. As we used FDA-approved drugs, the best-ranked molecules could be opportunities for drug repositioning with predicted antibacterial indication.

In addition to the above results, polypharmacology (multi-target) cases considering all selected drug targets were obtained using three frequency-based methods. Based on the first method that considers only the drugs in the top rank, those drugs that interact with more than 3 essential targets are listed in Table 3.

**Table 3:** Polypharmacology molecules selected based on the top rank of targets.

Molecule	Number of targets	Targets
ZINC000085537014	5	1WXI_AMP, 1Q7B_NAP, 5M29_CBY, 3KQJ_UD1, 1EQ2_NAP
ZINC000003914596	3	LI52_CTP, 5EJ8_TD6, 3ETH_ATP
ZINC000003941829	3	1GSA_ADP, 1GSA_GSH, 5ETP_5RZ
ZINC000085432544	3	2O1S_TDP, 1HV9_UD1, 5M1D_4LU

**Fig 2:**
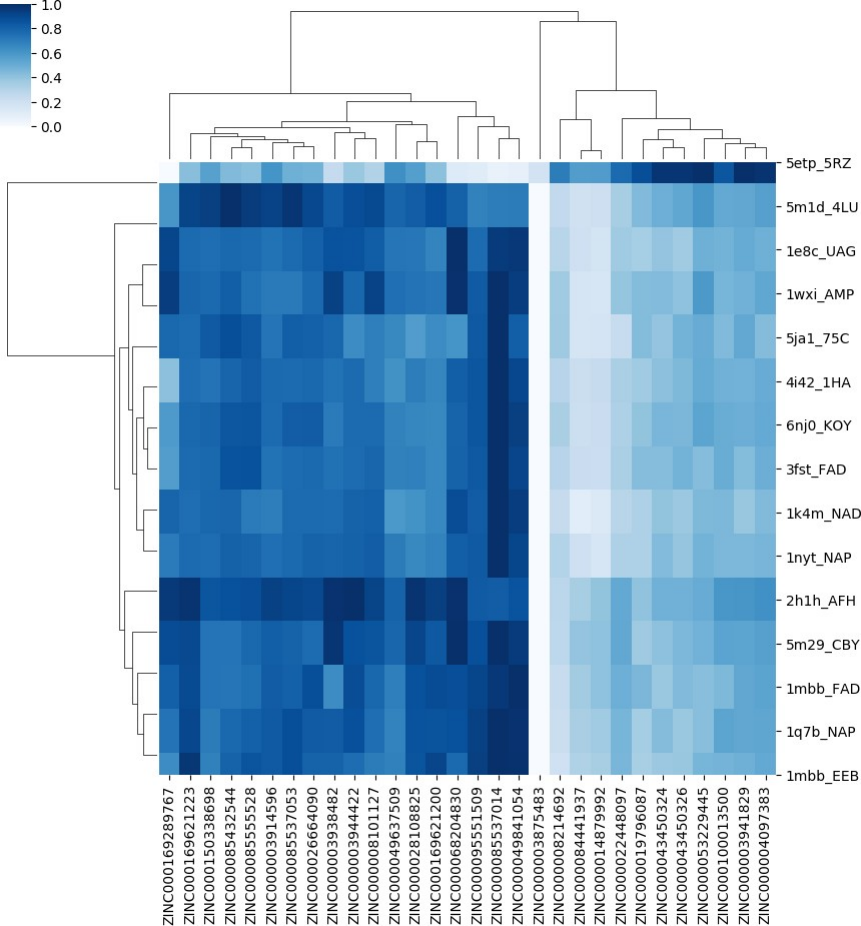
The heat map of selected ligands from the top five ranks of essential targets.

**Fig 3:**
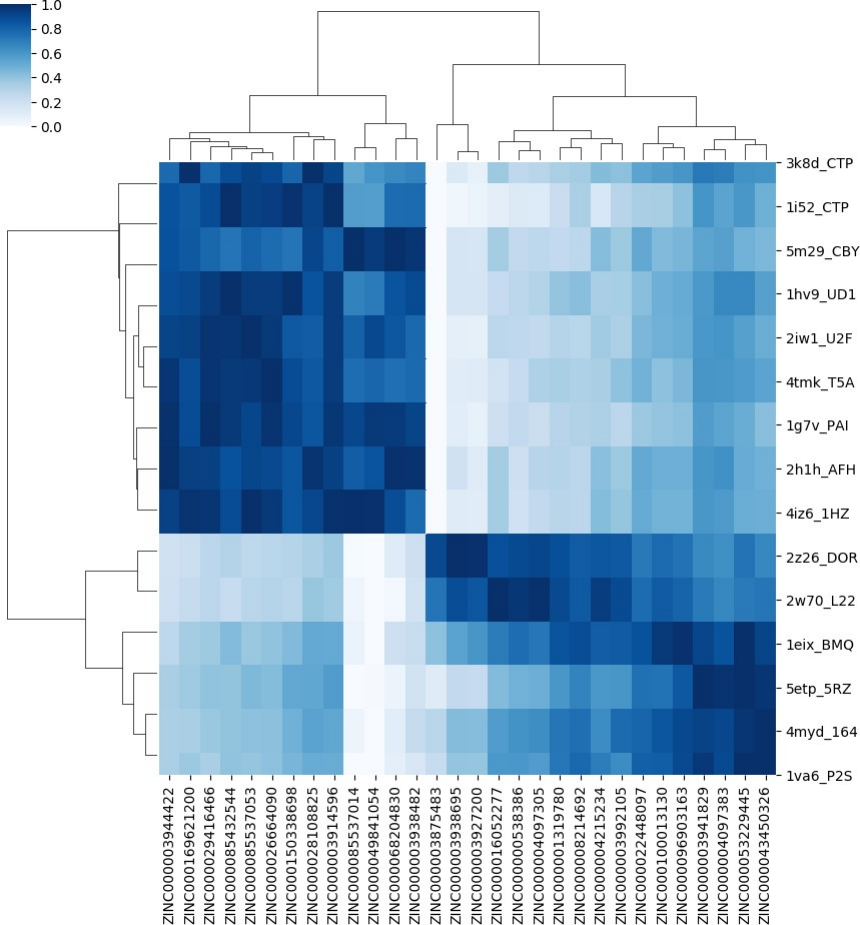
The heat map of selected ligands from the 1-percentile method.

The percentile-based approach is more suitable in cases where the scores of ranked molecules for each essential target are very close to each other and causes the loss of fewer good molecules. Figs 2 and 3 show the heat maps of selected ligands from the top five ranks and 1-percentile methods, respectively. The selected ligands are polypharmacology cases that can stop the growth of the pathogen using the inhibition of multiple drug targets.

The colorbar of the heatmaps ranges from white (assigned to the lowest normalized score values) to dark blue (assigned to the highest normalized score values) for each target. Thus, for each drug target, by moving row-wise on the heat map, one can find the best interacting ligand as the one with the darkest blue shade on that row.

The target dendrogram shows the hierarchical clustering of 92 protein-ligand pairs. This clustering is based on the similarity of the surface patches (which, in turn, is translated to similarity of the score rankings) of the selected drugs that are proposed as potentially active compounds for the pathogen. Enzymes are in the same cluster if they are similar in the binding ordering of these drugs. According to the ligand dendrogram, selected ligands are in the same cluster if their score values to the essential enzymes are similar. The labels displayed on the vertical axis of heat maps represent the top 15 targets to which the selected molecules have the strongest interactions. According to Fig 2, the cluster of 5etp is different from the cluster of the other 14 targets. Also, according to Fig 3, the cluster of 3K8D, LI52, 5M29, 1HV9, 2IW1, 4TMK, 1G7V, 2H1H, and 4IZ6 is different from the cluster of 2Z26, 2W70, 1EIX, 5ETP, 4MYD, and 1VA6 considering the selected ligand.

### 3.6. Investigation of ATC-code and safety of the selected drugs

We have presented drug IDs and ATC-codes of selected molecules (drugs) in Table 4. The first level of ATC-code is the anatomical main group and contains one letter. The second level shows the therapeutic subgroup and is two digits. The third level is the therapeutic/pharmacological subgroup and has one letter. The fourth level indicates the chemical/therapeutic/pharmacological subgroup and is one letter. The fifth level is the chemical substance and has two digits. According to WHO Guidelines 2020, anti-infectives are classified in J, A01AB, A02BD, A07A, D01, D06, D07C, D09AA, D10AF, G01, R02AB, and S01/S02/S03 groups [53]. Therefore, among the drugs in Table 4, Rifaximin (DB01220), Doxycycline (DB00254), Tobramycin (DB00684), Rifampicin (DB01045), Grazoprevir (DB11575), Tetracycline (DB00759), Minocycline (DB01017), Retapamulin (DB01256) are in the category of anti-infectives that have been suggested by our pipeline. It should be noted that, although the result is obtained based on integrating validated GEM with the validated SBVS method, they need to be tested experimentally. Also, we have extracted the ChEMBL IDs of the selected drugs from the UniChem database. Then, we have obtained the human genes (targets) related to these drugs via ChEMBL API (https://www.ebi.ac.uk/chembl) [54]. The ten drugs that have no known human targets are listed in Table 4. Among the selected drugs, Grazoprevir and Retapamulin are anti-infectives, and also they have no human targets. Also, there is a record of Retapamulin [55,56] in the treatment of bacterial infections in DrugBank. These results could be a sign of validation of our work. The compounds without any known human targets could be better options for experimental tests against *E*. *coli* infection.

**Table 4:** IDs and ATC-codes of the selected molecules.

ZINC ID	Drug Name	DrugBank ID	ATC-code	ChEMBL ID	Known Human Targets?	method
ZINC000003944422	Ritonavir	DB00503	J05AE03	CHEMBL163		2,3
ZINC000169621200	Rifaximin	DB01220	A07AA11/ D06AX11	CHEMBL1617		2,3
ZINC000029416466	Saquinavir	DB01232	J05AE01	CHEMBL296480	No	3
ZINC000085432544	Vinblastine	DB00570	L01CA01	CHEMBL159		1,2,3
ZINC000085537053	Docetaxel	DB01248	L01CD02	CHEMBL92		3
ZINC000026664090	Saquinavir	DB01232	J05AE01	CHEMBL296480	No	2,3
ZINC000150338698	Capreomycin	DB00314	J04AB30			2,3
ZINC000028108825	Gadofosveset trisodium	DB06705	V08CA11	CHEMBL1615469	No	2,3
ZINC000003914596	Saquinavir	DB01232	J05AE01	CHEMBL114		1,2,3
ZINC000085537014	Cobicistat	DB09065	J05AR09/J05AR15/J05AR14/J05AR22 /J05AR18 /V03AX03	CHEMBL2095208		1,2,3
ZINC000049841054	Carfilzomib	DB08889	L01XX45	CHEMBL451887		2,3
ZINC000068204830	Daclatasvir		J05AP58/J05AP07	CHEMBL2023898		2,3
ZINC000003938482	Posaconazole		J02AC04	CHEMBL1397		2,3
ZINC000003875483	Oxymorphone		N02AA11 (WHO)	CHEMBL963		2,3
ZINC000003938695	Norgestimate	DB00957	G03AA11	CHEMBL1200934		3
ZINC000003927200	Drospirenone	DB01395	G03AC10/G03FA17/G03AA12	CHEMBL1509		3
ZINC000016052277	Doxycycline	DB00254	J01AA02/A01AB22/J01AA20	CHEMBL1433		3
ZINC000000538386	Sufentanil	DB00708	N01AH03	CHEMBL658		3
ZINC000004097305	Flunisolide	DB00180	R01AD04/R03BA03	CHEMBL1512		3
ZINC000001319780	Buprenorphine	DB00921	N07BC01/N07BC51/N02AE01	CHEMBL560511		3
ZINC000008214692	Tobramycin	DB00684	S01AA12/J01GB01	CHEMBL1747		2,3
ZINC000004215234	Cefditoren	DB01066	J01DD16	CHEMBL1743	No	3
ZINC000003992105	Fluticasone furoate	DB08906	R03AL08/R01AD12/R03AK10/R03BA09	CHEMBL1676		3
ZINC000022448097	Gadoxetic acid	DB08884	V08CA10	CHEMBL2110606	No	2,3
ZINC000100013130	Midostaurin	DB06595	L01XE39	CHEMBL608533		3
ZINC000096903163	Diacetyl benzoyl lathyrol	DB11260		CHEMBL552128	No	3
ZINC000003941829	Fosamprenavir	DB01319	J05AE07	CHEMBL1664		1,2,3
ZINC000004097383	Pancuronium	DB01337	M03AC01	CHEMBL185073		2,3
ZINC000053229445	Rocuronium	DB00728	M03AC09	CHEMBL1201244		2,3
ZINC000043450326	Omacetaxine mepesuccinate	DB04865	L01XX40			2,3
ZINC000169289767	Trypan blue	DB09158		CHEMBL1089641		2
ZINC000169621223	Rifampicin	DB01045	J04AM02/J04AB02/J04AM06 /J04AM05/J04AM07	CHEMBL374478		2
ZINC000085555528	Vinblastine	DB00570	L01CA01	CHEMBL22969		2
ZINC000008101127	Indocyanine green	DB09374		CHEMBL1615807	No	2
ZINC000049637509	Isavuconazonium	DB06636	J02AC05	CHEMBL1183349	No	2
ZINC000095551509	Grazoprevir	DB11575	J05AP11/J05AP54	CHEMBL2063090	No	2
ZINC000084441937	Tetracycline	DB00759	S02AA08/A02BD02/A01AB13/J01AA07 /S01AA09/J01AA20 /J01RA08/S03AA02 /D06AA04 /A02BD08	CHEMBL1440		2
ZINC000014879992	Minocycline	DB01017	J01AA08/J01AA20 /A01AB23/	CHEMBL1434		2
ZINC000019796087	Nicardipine	DB00622	C08CA04	CHEMBL1598680		2
ZINC000043450324	Omacetaxine mepesuccinate	DB04865	L01XX40	CHEMBL175858		2
ZINC000100013500	Retapamulin	DB01256	D06AX13	CHEMBL1658	No	2

## 4. Conclusion

The discovery of novel antibacterial agents is necessary due to the rapid worldwide emergence of antibiotic resistance. GEMs are representative models of organisms at the metabolism level and they are good frameworks for the investigation of bacterial phenotypes. In this study, we have developed a structural systems pharmacology framework based on analyzing a metabolic network and a SBVS approach. The coupling of these two methods was done to achieve better results. As an example of its application, we have represented that this framework works well for *E*. *coli* and we could find anti-infective molecules for it. This can also be a general pipeline for the development of novel antibacterials for other bacterial pathogens that have GEM. Here, we have performed constraint-based flux analysis (FBA) on the most complete *E*. *coli* GEM-PRO for the rational and system-level identification of essential genes whose knocking out causes the growth of the pathogen to stop. 195 genes that are essential for the survival of the pathogen are identified and high-priority proteins related to these genes are detected as potential drug targets to carry out SBVS. These targets are the most promising candidates due to the availability of experimental structure in the PDB database and having cognate biologically relevant ligands. The SBVS method was performed with FDA-approved drugs for these targets by PLPS2. It evaluates interactions between a protein and a small molecule, based on molecular surfaces with shape, electrostatic potential, hydrophobicity, and hydrogen bonding features. Finally, we have identified new potential inhibitors among available FDA-approved drugs to stop the growth of the pathogen. Working with available drugs instead of other small molecules is an advantage because of the fast gaining drug resistance of pathogens. Therefore, the quicker discovery of new safe drugs is urgent. Here, we have predicted a new therapeutic indication (antibacterial) for reported drugs. Consequently, these drugs can be proposed as drug repositioning opportunities. It will be valuable if the proposed anti-infective drugs will be shifted to in vitro and in vivo experiments soon for validation of the results.

## Supporting information

S1 Table The components of the defined medium. (DOCX)

S2 Table Description and BP GO terms of identified essential genes that are in the “Cofactor and Prosthetic Group Biosynthesis” subsystem. (DOCX)

S1 File The list of essential genes and their UniProt IDs. (XLSX)

S2 File The complete list of the essential genes and their related information. (XLSX)

S3 File The ranked ligands against each identified target associated with their score values. (XLSX)

S1 Graphical Abstract (TIF)
