# Supplementary material for: Structural systems pharmacology: A framework for integrating metabolic network and structure-based virtual screening for drug discovery against bacteria
Source: PLoS One. 2021 Dec 14;16(12):e0261267. doi: 10.1371/journal.pone.0261267 (PMC8670682; doi:10.1371/journal.pone.0261267)
Supplement: S1 Table — (DOCX) [file pone.0261267.s001.docx]

| **Table S1. Components of the medium** | | |
| --- | --- | --- |
| **Component** | **Reaction** | **Flux lower bound** |
| Adenosylcobalamin | EX_adocbl_e | -0.1 |
| L-Alanine | EX_ala__L_e | -0.1 |
| L-Arginine | EX_arg__L_e | -0.1 |
| L-Aspartate | EX_asp__L_e | -0.1 |
| Biotin | EX_btn_e | -0.1 |
| Calcium | EX_ca2_e | -1000 |
| Choline | EX_chol_e | -0.1 |
| Chloride | EX_cl_e | -1000 |
| CO2 | EX_co2_e | -1000 |
| Co2+ | EX_cobalt2_e | -1000 |
| Cu2+ | EX_cu2_e | -1000 |
| L-Cysteine | EX_cys__L_e | -0.1 |
| Fe2+ | EX_fe2_e | -1000 |
| Fe3+ | EX_fe3_e | -1000 |
| D-Glucose | EX_glc__D_e | -10 |
| L-Glutamate | EX_glu__L_e | -0.1 |
| Glycine | EX_gly_e | -0.1 |
| H2O | EX_h2o_e | -1000 |
| H+ | EX_h_e | -1000 |
| L-Histidine | EX_his__L_e | -0.1 |
| L-Isoleucine | EX_ile__L_e | -0.1 |
| myo-Inositol | EX_inost_e | -0.1 |
| potassium | EX_k_e | -1000 |
| L-Leucine | EX_leu__L_e | -0.1 |
| L-Lysine | EX_lys__L_e | -0.1 |
| L-Methionine | EX_met__L_e | -0.1 |
| magnesium | EX_mg2_e | -1000 |
| Mn2+ | EX_mn2_e | -1000 |
| Molybdate | EX_mobd_e | -1000 |
| Sodium | EX_na1_e | -1000 |
| Ammonium | EX_nh4_e | -1000 |
| nickel | EX_ni2_e | -1000 |
| O2 | EX_o2_e | -1000 |
| Ornithine | EX_orn_e | -0.1 |
| L-Phenylalanine | EX_phe__L_e | -0.1 |
| Phosphate | EX_pi_e | -1000 |
| (R)-Pantothenate | EX_pnto__R_e | -0.1 |
| L-Proline | EX_pro__L_e | -0.1 |
| Pyridoxal | EX_pydx_e | -0.1 |
| Selenate | EX_sel_e | -1000 |
| L-Serine | EX_ser__L_e | -0.1 |
| selenite | EX_slnt_e | -1000 |
| Sulfate | EX_so4_e | -1000 |
| Taurine | EX_taur_e | -0.1 |
| Thiamin | EX_thm_e | -0.1 |
| L-Threonine | EX_thr__L_e | -0.1 |
| L-Tryptophan | EX_trp__L_e | -0.1 |
| tungstate | EX_tungs_e | -1000 |
| L-Tyrosine | EX_tyr__L_e | -0.1 |
| L-Valine | EX_val__L_e | -0.1 |
| Zinc | EX_zn2_e | -1000 |
