# Supplementary material for: Structural systems pharmacology: A framework for integrating metabolic network and structure-based virtual screening for drug discovery against bacteria
Source: PLoS One. 2021 Dec 14;16(12):e0261267. doi: 10.1371/journal.pone.0261267 (PMC8670682; doi:10.1371/journal.pone.0261267)
Supplement: S2 Table — (DOCX) [file pone.0261267.s002.docx]

| **Table S2. Essential genes involved in Cofactor and Prosthetic Group Biosynthesis subsystem** | | | | |
| --- | --- | --- | --- | --- |
| **Essential genes** | **UniProt IDs** | **Gene description** | **BP^*^ GO term IDs** | **GO Terms** |
| b3368 | P0AEA8 | siroheme synthase | GO:0009236 | cobalamin biosynthetic process |
|  |  |  | GO:0019354 | siroheme biosynthetic process |
| b0414 | P25539 | fused diaminohydroxyphosphoribosylaminopyrimidine deaminase/5-amino-6-(5-phosphoribosylamino)uracil reductase | GO:0009231 | riboflavin biosynthetic process |
| b3041 | P0A7J0 | 3,4-dihydroxy-2-butanone-4-phosphate synthase | GO:0009231 | riboflavin biosynthetic process |
| b0025 | P0AG40 | bifunctional riboflavin kinase/FMN adenylyltransferase | GO:0006747 | FAD biosynthetic process |
|  |  |  | GO:0009398 | FMN biosynthetic process |
|  |  |  | GO:0009231 | riboflavin biosynthetic process |
| b1662 | P0AFU8 | riboflavin synthase | GO:0009231 | riboflavin biosynthetic process |
| b0421 | P22939 | geranyl diphosphate/farnesyl diphosphate synthase | GO:0045337 | farnesyl diphosphate biosynthetic process |
|  |  |  | GO:0033384 | geranyl diphosphate biosynthetic process |
| b3804 | P09126 | uroporphyrinogen-III synthase | GO:0006782 | protoporphyrinogen IX biosynthetic process |
|  |  |  | GO:0006780 | uroporphyrinogen III biosynthetic process |
| b3997 | P29680 | uroporphyrinogen decarboxylase | GO:0006783 | heme biosynthetic process |
|  |  |  | GO:0006779 | porphyrin-containing compound biosynthetic process |
|  |  |  | GO:0019353 | protoporphyrinogen IX biosynthetic process from glutamate |
|  |  |  | GO:0006780 | uroporphyrinogen III biosynthetic process |
| b0639 | P0A752 | nicotinate-mononucleotide adenylyltransferase | GO:0034628 | de novo NAD biosynthetic process from aspartate |
|  |  |  | GO:0009435 | NAD biosynthetic process |
|  |  |  | GO:0034355 | NAD salvage |
| b0415 | P61714 | 6,7-dimethyl-8-ribityllumazine synthase | GO:0009231 | riboflavin biosynthetic process |
| b0174 | P60472 | ditrans,polycis-undecaprenyl-diphosphate synthase [(2E,6E)-farnesyl-diphosphate specific] | GO:0016094 | polyprenol biosynthetic process |
| b3639 | P0ABQ0 | fused 4'-phosphopantothenoylcysteine decarboxylase and phosphopantothenoylcysteine synthetase | GO:0015937 | coenzyme A biosynthetic process |
| b1210 | P0A6X1 | glutamyl-tRNA reductase | GO:0019353 | protoporphyrinogen IX biosynthetic process from glutamate |
| b0428 | P0AEA5 | heme O synthase | GO:0006783 | heme biosynthetic process |
|  |  |  | GO:0048034 | heme O biosynthetic process |
| b0154 | P23893 | glutamate-1-semialdehyde 2,1-aminomutase | GO:0006779 | porphyrin-containing compound biosynthetic process |
|  |  |  | GO:0006782 | protoporphyrinogen IX biosynthetic process |
|  |  |  | GO:0033014 | tetrapyrrole biosynthetic process |
| b2232 | P17993 | bifunctional 3-demethylubiquinone-8 3-O-methyltransferase and 2-octaprenyl-6-hydroxyphenol methylase | GO:0006744 | ubiquinone biosynthetic process |
| b0142 | P26281 | 2-amino-4-hydroxy-6-hydroxymethyldihydropteridine diphosphokinase | GO:0046656 | folic acid biosynthetic process |
|  |  |  | GO:0046654 | tetrahydrofolate biosynthetic process |
| b3634 | P0A6I6 | pantetheine-phosphate adenylyltransferase | GO:0015937 | coenzyme A biosynthetic process |
| b0173 | P45568 | 1-deoxy-D-xylulose 5-phosphate reductoisomerase | GO:0019288 | isopentenyl diphosphate biosynthetic process, methylerythritol 4-phosphate pathway |
|  |  |  | GO:0051484 | isopentenyl diphosphate biosynthetic process, methylerythritol 4-phosphate pathway involved in terpenoid biosynthetic process |
| b3843 | P0AAB4 | 3-octaprenyl-4-hydroxybenzoate decarboxylase | GO:0006744 | ubiquinone biosynthetic process |
|  |  |  | GO:0032150 | ubiquinone biosynthetic process from chorismate |
| b2615 | P0A7B3 | NAD kinase | GO:0006741 | NADP biosynthetic process |
| b2261 | P29208 | o-succinylbenzoate synthase | GO:0009234 | menaquinone biosynthetic process |
| b0596 | P15047 | 2,3-dihydro-2,3-dihydroxybenzoate dehydrogenase | GO:0009239 | enterobactin biosynthetic process |
| b0595 | P0ADI4 | enterobactin synthase component B | GO:0009239 | enterobactin biosynthetic process |
| b0420 | P77488 | 1-deoxy-D-xylulose-5-phosphate synthase | GO:0008615 | pyridoxine biosynthetic process |
|  |  |  | GO:0016114 | terpenoid biosynthetic process |
|  |  |  | GO:0009228 | thiamine biosynthetic process |
|  |  |  | GO:0006744 | ubiquinone biosynthetic process |
| b1208 | P62615 | 4-(cytidine 5'-diphospho)-2-C-methyl-D-erythritol kinase | GO:0016114 | terpenoid biosynthetic process |
| b2746 | P62617 | 2-C-methyl-D-erythritol 2,4-cyclodiphosphate synthase | GO:0016114 | terpenoid biosynthetic process |
|  |  |  | GO:0006744 | ubiquinone biosynthetic process |
| b1277 | P0A7I7 | GTP cyclohydrolase 2 | GO:0009231 | riboflavin biosynthetic process |
| b0109 | P30011 | quinolinate phosphoribosyltransferase (decarboxylating) | GO:0034628 | de novo' NAD biosynthetic process from aspartate |
|  |  |  | GO:0009435 | de novo' NAD biosynthetic process from aspartate |
| b3187 | P0AD57 | all-trans-octaprenyl-diphosphate synthase | GO:0016094 | polyprenol biosynthetic process |
|  |  |  | GO:0006744 | ubiquinone biosynthetic process |
| b0586 | P11454 | apo-serine activating enzyme | GO:0009239 | enterobactin biosynthetic process |
| b4040 | P0AGK1 | 4-hydroxybenzoate octaprenyltransferase | GO:0006744 | ubiquinone biosynthetic process |
| b3974 | P0A6I3 | pantothenate kinase | GO:0015937 | coenzyme A biosynthetic process |
| b0103 | P0A6I9 | dephospho-CoA kinase | GO:0015937 | coenzyme A biosynthetic process |
| b1812 | P05041 | aminodeoxychorismate synthase subunit 1 | GO:0046656 | folic acid biosynthetic process |
|  |  |  | GO:0046654 | tetrahydrofolate biosynthetic process |
|  |  |  | GO:0008153 | para-aminobenzoic acid biosynthetic process |
| b0029 | P62623 | 1-hydroxy-2-methyl-2-(E)-butenyl 4-diphosphate reductase | GO:0016114 | terpenoid biosynthetic process |
| b0417 | P0AGG0 | thiamine monophosphate kinase | GO:0009228 | thiamine biosynthetic process |
|  |  |  | GO:0009229 | thiamine diphosphate biosynthetic process |
| b0583 | P19925 | phosphopantetheinyl transferase EntD | GO:0009237 | siderophore metabolic process |
| b2688 | P0A6W9 | glutamate--cysteine ligase | GO:0006750 | glutathione biosynthetic process |
| b1740 | P18843 | NH3-dependent NAD(+) synthetase | GO:0034628 | de novo' NAD biosynthetic process from aspartate |
|  |  |  | GO:0009435 | NAD biosynthetic process |
|  |  |  | GO:0034355 | NAD salvage |
| b0369 | P0ACB2 | porphobilinogen synthase | GO:0006783 | heme biosynthetic process |
| b2153 | P0A6T5 | GTP cyclohydrolase 1 | GO:0035998 | 7,8-dihydroneopterin 3'-triphosphate biosynthetic process |
|  |  |  | GO:0006729 | tetrahydrobiopterin biosynthetic process |
|  |  |  | GO:0008616 | queuosine biosynthetic process |
|  |  |  | GO:0046654 | tetrahydrofolate biosynthetic process |
| b2315 | P08192 | bifunctional folylpolyglutamate synthetase/dihydrofolate synthetase | GO:0046656 | folic acid biosynthetic process |
|  |  |  | GO:0046654 | tetrahydrofolate biosynthetic process |
| b2574 | P10902 | L-aspartate oxidase | GO:0034628 | de novo' NAD biosynthetic process from aspartate |
| b3177 | P0AC13 | dihydropteroate synthase | GO:0046654 | tetrahydrofolate biosynthetic process |
|  |  |  | GO:0046656 | folic acid biosynthetic process |
| b0750 | P11458 | quinolinate synthase | GO:0034628 | de novo' NAD biosynthetic process from aspartate |
| b3805 | P06983 | hydroxymethylbilane synthase | GO:0006783 | heme biosynthetic process |
|  |  |  | GO:0006782 | protoporphyrinogen IX biosynthetic process |
|  |  |  | GO:0033014 | tetrapyrrole biosynthetic process |
| b2747 | Q46893 | 2-C-methyl-D-erythritol 4-phosphate cytidylyltransferase | GO:0016114 | terpenoid biosynthetic process |
| b2947 | P04425 | glutathione synthetase | GO:0006750 | glutathione biosynthetic process |
| b3850 | P0ACB4 | protoporphyrinogen oxidase | GO:0006783 | heme biosynthetic process |
|  |  |  | GO:0006779 | porphyrin-containing compound biosynthetic process |
|  |  |  | GO:0006782 | protoporphyrinogen IX biosynthetic process |
| b2260 | P37353 | o-succinylbenzoate--CoA ligase | GO:0009234 | menaquinone biosynthetic process |
| b0594 | P10378 | 2,3-dihydroxybenzoate-AMP ligase | GO:0009239 | enterobactin biosynthetic process |
| b3833 | P0A887 | bifunctional 2-octaprenyl-6-methoxy-1,4-benzoquinol methylase and demethylmenaquinone methyltransferase | GO:0009234 | menaquinone biosynthetic process |
|  |  |  | GO:0006744 | ubiquinone biosynthetic process |
| b3930 | P32166 | 1,4-dihydroxy-2-naphthoate octaprenyltransferase | GO:0009234 | menaquinone biosynthetic process |
|  |  |  | GO:0032194 | ubiquinone biosynthetic process via 3,4-dihydroxy-5-polyprenylbenzoate |
|  |  |  | GO:0042371 | vitamin K biosynthetic process |
| b0159 | P0AF12 | 5'-methylthioadenosine/S-adenosylhomocysteine nucleosidase | GO:0019509 | L-methionine salvage from methylthioadenosine |
|  |  |  | GO:0019284 | L-methionine salvage from S-adenosylmethionine |
| b1093 | P0AEK2 | 3-oxoacyl-[acyl-carrier-protein] reductase FabG | GO:0009102 | biotin biosynthetic process |
| b0009 | P0AF03 | molybdopterin adenylyltransferase | GO:0032324 | molybdopterin cofactor biosynthetic process |
|  |  |  | GO:0006777 | Mo-molybdopterin cofactor biosynthetic process |
| b0784 | P30748 | molybdopterin synthase sulfur carrier subunit | GO:0006777 | Mo-molybdopterin cofactor biosynthetic process |
| b2530 | P0A6B7 | cysteine desulfurase IscS | GO:0018131 | oxazole or thiazole biosynthetic process |
| b0827 | P12281 | molybdopterin molybdotransferase | GO:0032324 | molybdopterin cofactor biosynthetic process |
|  |  |  | GO:0006777 | Mo-molybdopterin cofactor biosynthetic process |
| b3857 | P32173 | molybdenum cofactor guanylyltransferase | GO:1902758 | bis(molybdopterin guanine dinucleotide)molybdenum biosynthetic process |
| b3058 | P0AC16 | dihydroneopterin aldolase | GO:0046656 | folic acid biosynthetic process |
|  |  |  | GO:0046654 | tetrahydrofolate biosynthetic process |
| b2263 | P37355 | 2-succinyl-6-hydroxy-2,4-cyclohexadiene-1-carboxylate synthase | GO:0009234 | menaquinone biosynthetic process |
| b2264 | P17109 | 2-succinyl-5-enolpyruvyl-6-hydroxy-3-cyclohexene-1-carboxylate synthase | GO:0009234 | menaquinone biosynthetic process |
| b2262 | P0ABU0 | 1,4-dihydroxy-2-naphthoyl-CoA synthase | GO:0009234 | menaquinone biosynthetic process |
| b1686 | P77781 | 1,4-dihydroxy-2-naphthoyl-CoA hydrolase | GO:0009234 | menaquinone biosynthetic process |
| b2877 | Q46810 | molybdenum cofactor cytidylyltransferase | GO:1902760 | Mo(VI)-molybdopterin cytosine dinucleotide biosynthetic process |
|  |  |  | GO:0006777 | Mo-molybdopterin cofactor biosynthetic process |
| b0785 | P30749 | molybdopterin synthase catalytic subunit | GO:0006777 | Mo-molybdopterin cofactor biosynthetic process |
| b2515 | P62620 | (E)-4-hydroxy-3-methylbut-2-enyl-diphosphate synthase (flavodoxin) | GO:0016114 | terpenoid biosynthetic process |
| b0826 | P12282 | molybdopterin-synthase adenylyltransferase | GO:0006777 | Mo-molybdopterin cofactor biosynthetic process |
| b0783 | P0A738 | cyclic pyranopterin monophosphate synthase | GO:0006777 | Mo-molybdopterin cofactor biosynthetic process |
| b0628 | P60716 | lipoyl synthase | GO:0009107 | lipoate biosynthetic process |
| b2907 | P25534 | 2-octaprenyl-6-methoxyphenol 4-hydroxylase | GO:0006744 | ubiquinone biosynthetic process |
| * Biological Process | | | | |
