# Supplementary figures and images for: Structural systems pharmacology: A framework for integrating metabolic network and structure-based virtual screening for drug discovery against bacteria
Source: PLoS One. 2021 Dec 14;16(12):e0261267. doi: 10.1371/journal.pone.0261267 (PMC8670682; doi:10.1371/journal.pone.0261267)

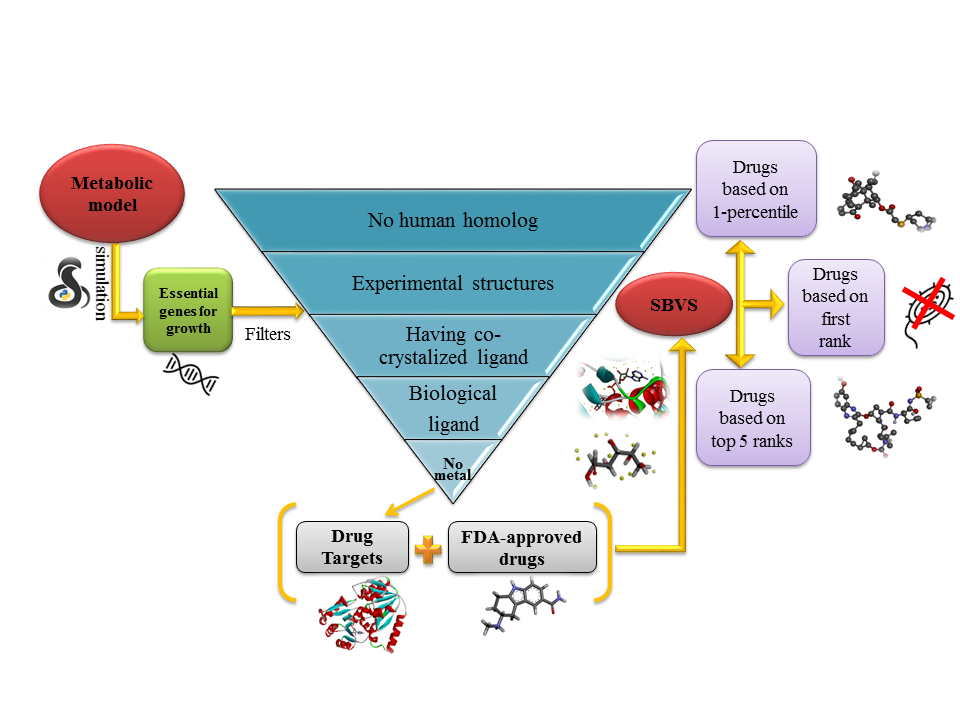

Supplement: S1 Graphical Abstract — (TIF) [file pone.0261267.s006.tif]
